# Matrix-Dependent Performance of β‑Glucan Composite Films Functionalized with Phenolics from Passion Fruit Seed Waste

**DOI:** 10.1021/acsomega.6c07059

**Published:** 2026-08-11

**Authors:** Guilherme Felipe Xavier, Gessica Maria Lopes Faria, Eric Keven Silva, Melina Savioli Lopes

**Affiliations:** † Programa de Pós-Graduação em Engenharia Química, Instituto de Ciência e Tecnologia, 74347 Universidade Federal de Alfenas (UNIFAL/MG), Campus Poços de Caldas, Poços de Caldas, Minas Gerais 37715-400, Brazil; ‡ Universidade Estadual de Campinas (UNICAMP), Faculdade de Engenharia de Alimentos (FEA), Rua Monteiro Lobato, 80, Campinas, São Paulo CEP 13083-862, Brazil

## Abstract

The valorization of agro-industrial waste for producing sustainable and functional packaging is a key strategy within the circular economy and green chemistry. This study developed active antioxidant edible films by incorporating a phenolic-rich extract from yellow passion fruit seeds into two composite polysaccharide matrices: β-glucan/carboxymethyl cellulose (BG/CMC) and β-glucan/citrus pectin (BG/PEC). The phenolic extract, obtained through a sequential ultrasound-assisted extraction from defatted yellow passion fruit seeds, was incorporated into the film-forming solutions at different concentrations (0, 57, 114, and 190 μg GAE/mL), which were subsequently cast and dried. The films were thoroughly characterized. Both film series exhibited excellent UV light barrier properties and homogeneous morphology. The BG/CMC films were fully water-soluble and showed the highest antioxidant activity (up to 19.42 μmol TE/g by TEAC assay) at the highest extract concentration. In contrast, BG/PEC films demonstrated superior and enhanced mechanical properties when loaded with the highest extract amount, achieving a balanced combination of tensile strength (3.64 MPa) and elongation at break (20.84%). Structural analyses (FTIR, XRD, and TGA) confirmed the successful incorporation of the extract and revealed an amorphous structure with good thermal stability. The BG/PEC films showed visible enhancement on thermal stability with the incorporation of the phenolic extract, in any concentration, with a final mass loss near 30%. All films showed good synergy within the matrices ingredients, corroborated by the FTIR spectra where the main bands were still present. The results demonstrate that the choice of the polysaccharide matrix (CMC vs pectin) dictates the final functional profile of the active film. This work successfully transforms passion fruit processing waste into tailorable, antioxidant edible films, providing sustainable alternatives for active food packaging applications.

## Graphical abstract

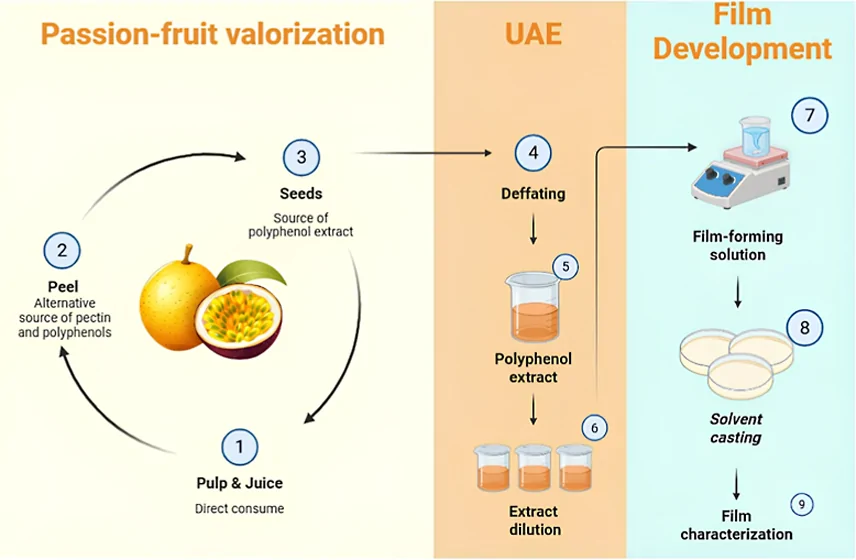

## 1 Introduction

The global demand for sustainable food packaging has intensified research into biodegradable and edible films derived from natural polymers. These materials provide a promising alternative to conventional petroleum-based plastics, reducing environmental pollution and aligning with circular economy principles.^1^ Among natural polymers, polysaccharides such as pectin, carboxymethyl cellulose (CMC), and β-glucan (BG) are particularly attractive due to their biodegradability, biocompatibility, and film-forming ability.^2,3,62^ However, films based on a single polysaccharide often exhibit significant limitations, such as poor water vapor barrier properties and inadequate mechanical strength, which restrict their practical application.^4,5^ To overcome these drawbacks, the development of composite films has emerged as a strategic approach, where the combination of different biopolymers can yield materials with tailored and enhanced properties.

The pursuit of sustainable raw materials has directed attention to agro-industrial wastes as abundant, low-cost sources of bioactive compounds and functional biopolymers. In this context, yellow passion fruit (*Passiflora edulis f. flavicarpa*) is a crop of great relevance, especially in Brazil, the world’s largest producer. The industrial processing of passion fruit for juice and pulp generates substantial waste, primarily peels and seeds, which constitute over 60% of the fruit’s total mass.^6^ The peels are rich in dietary fibers and polysaccharides like pectin, while the seeds contain significant levels of phenolic compounds, lipids, and other bioactive molecules with demonstrated antioxidant activity.^7,57^ Valorizing these wastes aligns with the principles of circular economy, transforming a disposal problem into a source of high-value ingredients for functional materials.^8^

Pectin, a complex heteropolysaccharide, is widely used as a gelling and stabilizing agent in the food industry. Commercial pectin is primarily extracted from citrus peel and apple pomace, creating a need to diversify its sources.^9^ Passion fruit peel has been identified as a potential alternative pectin source. However, pectin-based films alone are highly hydrophilic and possess limited water resistance and mechanical properties.^10^ Similarly, films based solely on CMC, despite excellent film-forming ability, often lack the necessary mechanical robustness and functional properties like intrinsic antioxidant activity.^11^ β-Glucan, a polysaccharide known for its bioactive properties and good film-forming capacity, can act as an effective complement in polymeric blends. Composite films of β-glucan with CMC or pectin have shown potential to improve mechanical strength, thermal stability, and barrier properties.^3,12^ Furthermore, the incorporation of bioactive extracts into edible films can transform them from simple barriers into active packaging systems. Active packaging interacts with the food product or its environment to extend shelf life, for instance, by providing antioxidant or antimicrobial activity.^13,58,64^ Phenolic compounds extracted from plant sources are excellent candidates for this purpose due to their potent antioxidant capacity.^14^ However, long-term stability of these phenolic compounds within polymer matrices is a critical factor determining their effectiveness as active packaging agents.^54^

Pectin and carboxymethylcellulose (CMC) were selected as the primary film-forming polymers for the following reasons: (i) both are polysaccharides capable of forming hydrogen bonds interactions with polar compounds (e.g., phenolic –OH groups and β-glucan), which is essential in the development of a homogeneous composite film;^1,2,4^ (ii) they differ in physical, thermal, and mechanical properties, allowing tunable formulations based on target application; and (iii) pectin and CMC represent a complementary approach in valorizing passion fruit processing waste: the richness of pectin in the passion fruit peel as well as the increasing search for different sources of pectin^2,6^ and the well-studied, commercial availability of CMC, serving as a benchmark for comparison.

The formulated matrices were functionalized by incorporating a phenolic-rich extract obtained from yellow passion fruit seeds via a sequential ultrasound-assisted extraction (UAE) process, which is known to enhance phenolic yield compared to conventional solvent extraction.^51,53^ While the incorporation of plant-derived phenolic compounds into polysaccharide films has been reported, the present work offers distinct novel contributions: (i) the use of *P. edulis* (passion fruit) seed extract instead of its most voluminous parts (e.g., pulp and peel) and (ii) a direct comparison of pectin versus CMC as the primary film-forming polymer under identical extract and β-glucan contents, while most literature reports only one type of polysaccharide blend data.^41,42,45,67^ The films were thoroughly characterized for their physical, morphological, structural, thermal, mechanical, and functional antioxidant properties.

Therefore, this study aimed to develop and characterize novel active edible films by combining the structural benefits of biopolymer blends with the functionality of a natural antioxidant extract obtained from yellow passion fruit seeds. The specific objectives are (i) to evaluate the effect of the polymer matrix (pectin vs CMC) on the physical, mechanical, thermal, structural, and barrier properties of the films; (ii) to assess the impact of different concentrations of the phenolic extract (0, 57, 114, and 190 μg GAE/mL) on film functionality; and (iii) to determine the antioxidant potential of the developed films using ferric reducing antioxidant power (FRAP) and Trolox equivalent antioxidant capacity (TEAC) assays. This work demonstrates a sustainable and integrated approach to valorizing passion fruit processing waste, converting it into functional, bioactive packaging materials with potential application in the food industry.

## 2 Materials and Methods

### 2.1 Raw Materials and Chemicals

Yellow passion fruits (*P. edulis f. flavicarpa*) were purchased from a local market in Poços de Caldas, MG, Brazil. Glycerol (analytical grade) was acquired from Synth (Diadema, SP, Brazil). Carboxymethyl cellulose (CMC, degree of substitution 0.9), 2,2′-azinobis­(3-ethylbenzothiazoline-6-sulfonic acid)­diammonium salt (ABTS), Folin–Ciocalteu reagent, 3,4,5-trihydroxybenzoic acid (gallic acid), and 6-hydroxy-2,5,7,8-tetramethylchroman-2-carboxylic acid (Trolox) were obtained from Sigma-Aldrich (Cotia, SP, Brazil). Citrus pectin (PEC) was purchased from Adicel (Belo Horizonte, MG, Brazil). Oat β-glucan (BG) was acquired from Nutramax (Catanduva, SP, Brazil). All other solvents and chemicals were of analytical grade.

### 2.2 UAE of Phenolic Compounds from Passion Fruit Seeds

UAE has been widely recognized as an effective alternate method for the extraction of phenolic compounds from plant-derived sources, offering good compatibility, increased yield, and reduced extraction time.^51,52,60,61,63^

Passion fruit seeds were separated from the pulp, washed, dried at room temperature, and ground. A sequential extraction of oil followed by phenolic compounds was performed using an ultrasonic processor VCX 750 (Sonics & Materials, Inc., USA), comprising a power generator and a transducer coupled to a 13 mm probe, operating at 20 kHz with a nominal power of 750 W and adjustable amplitude (20–100%). In the first stage, the oil fraction was extracted using absolute ethanol (solid-to-solvent ratio of 1:20 w/v) at 80% amplitude for 10 min per cycle over five cycles. The resulting defatted solid residue was then subjected to phenolic extraction using 50% (v/v) aqueous ethanol under the same solid-to-solvent ratio and amplitude conditions. This second extraction stage was conducted for a total of 60 min with solvent renewal at predefined intervals (2.5, 5, 10, 15, 20, 30, 40, 50, and 60 min). The extracts corresponding to the intervals between 2.5 and 20 min were pooled to obtain the final phenolic-rich extract used for the film formulation.

The total phenolic content (TPC) of the extracts was determined by the Folin–Ciocalteu spectrophotometric method,^15^ with slight modifications. For the assays, 25 μL of extract samples was added to 25 μL of Folin–Ciocalteu (50% v/v) and 200 μL of Na_2_CO_3_ (5% w/v) solution. The microplates were incubated for 20 min in a dark environment before the absorbance reading at 760 nm. A calibration curve (*R* ^2^ = 0.997) using standard gallic acid as a reference was used. The results were expressed as μg gallic acid equivalents (GAE) per mL of extract.

### 2.3 Edible Film Production by Solvent Casting

Edible films were prepared using the solvent casting technique. Two series of composite films were developed: one based on a blend of BG and CMC and another based on a blend of BG and commercial citrus pectin (PEC). For each series, the aqueous phase of the film-forming solution consisted of distilled water (control) or the passion fruit seed phenolic extract at different concentrations.

The film-forming solutions had the following fixed composition (w/w): 1.5% BG, 1.5% structuring polymer (CMC or PEC), 1.2% glycerol (plasticizer), and 95.8% aqueous phase. The aqueous phase was adjusted to provide final GAE concentrations in the film solution of approximately 0, 57, 114, and 190 μg GAE/mL, corresponding to passion fruit seed extract dilutions of 0% (E_0_, control), 30% (E_30_), 60% (E_60_), and 100% (E_100_), respectively. The detailed formulations are summarized in Table 1.

**Table 1:** Formulation (% w/w) of β-Glucan (BG) Composite Films Containing Carboxymethylcellulose (CMC) or Pectin (PEC) and Different Concentrations of Phenolic Extract

edible film	CMC	PEC	BG	glycerol	aqueous phase	phenolic concentration (μg GAE/mL)
CMC/E_0_	1.5	0	1.5	1.2	95.8	0
CMC/E_30_	1.5	0	1.5	1.2	95.8	57
CMC/E_60_	1.5	0	1.5	1.2	95.8	114
CMC/E_100_	1.5	0	1.5	1.2	95.8	190
PEC/E_0_	0	1.5	1.5	1.2	95.8	0
PEC/E_30_	0	1.5	1.5	1.2	95.8	57
PEC/E_60_	0	1.5	1.5	1.2	95.8	114
PEC/E_100_	0	1.5	1.5	1.2	95.8	190

The biopolymers were dispersed in the aqueous phase under magnetic stirring at 400 rpm and heated to 80 °C for 30 min. Eventual volume loss by evaporation was compensated by adding distilled water in the solution, followed by homogenization. Then, 23 g of the film-forming solution was cast onto Petri dishes (9 cm diameter) and dried in a forced-air oven at 40 °C for 24 h. The dried films were carefully peeled off and conditioned in a desiccator containing silica gel at 25 °C and 50% relative humidity for at least 48 h before characterization.

### 2.4 Edible Film Characterization

#### 2.4.1 Thickness and Color

The film thickness was measured at four random positions using a digital caliper rule (0.01 mm resolution). Color parameters (*L*, *a*, b*) were determined using a portable colorimeter (CR-400, Konica Minolta, Japan) based on the CIE Lab system, with a white standard plate as the background. Measurements were performed in quintuplets.

#### 2.4.2 Morphology

The surface and cross-sectional morphologies of the films were examined by scanning electron microscopy (SEM) using a LEO 440i microscope (LEO Electron Microscopy, Germany). Samples were coated with a gold/palladium mixture before analysis for 45 s, at a current of 20 mA. SEM was performed with magnifications between 500 and 1000 times.

#### 2.4.3 Optical Properties

The light barrier properties of the films were evaluated by ultraviolet–visible (UV–vis) spectroscopy in the range of 200–1000 nm using a spectrophotometer. Scanning was performed in 3 different portions of the films. The transmittance was recorded to assess UV and visible light barrier capacity, respectively.

#### 2.4.4 Water Solubility and Water Vapor Permeability

Water solubility (WS) was determined according to the method described by Nahas, Andrade, Lopes, and Silva,^16^ with modifications. Film samples (2 × 2 cm) were dried at 105 °C for 12 h to obtain the initial dry weight (*W*_i_). They were then immersed in 50 mL of distilled water under constant agitation for 1 h at 25 °C. The insoluble residue was recovered by filtration, dried again at 105 °C for 12 h, and weighed (*W*_f_). WS was calculated according to eq 1:

1 $$WS\;\left(\%\right)=\frac{W_{i}-W_{f}}{W_{i}}\times 100$$

Water vapor permeability (WVP) was determined gravimetrically according to the ASTM E96-00 standard method with modifications. Films were sealed over glass cups containing anhydrous calcium chloride (0% relative humidity) and placed in a desiccator maintained at 25 °C and 100% relative humidity (distilled water). The weight gain of the cup was recorded at 1 h intervals over 8 h. WVP (g·m^–1^·s^–1^·Pa^–1^) was calculated from the steady-state slope of weight gain versus time, the film area, the vapor pressure difference, and the mean film thickness. All measurements were performed in triplicate.

#### 2.4.5 Structural and Thermal Characterization

For the crystallinity, the X-ray diffraction (XRD) patterns were recorded using a Rigaku DMAX diffractometer (Rigaku Corp., Japan) at 30 kV, in the 2θ range of 4–60° with an increment of 0.02°. Fourier transform infrared (FTIR) spectroscopy spectra were obtained using an Agilent Cary 630 FTIR Spectrometer (Agilent Technologies, USA) in the range of 4000–600 cm^–1^ with a resolution of 2 cm^–1^. Thermal stability (TGA) was evaluated using a Netzsch STA 449F3 Jupiter instrument (Netzsch-Gerätebau GmbH, Germany), where samples of 2 cm × 2 cm were heated from 28 to 600 °C at a rate of 10 °C/min under a nitrogen atmosphere.

#### 2.4.6 Mechanical Properties

Tensile strength (TS) and elongation at break (EAB) were determined using a texture analyzer (TAXTplus, TA Instruments, USA) according to ASTM D882. Film strips (30 mm × 10 mm) were conditioned at 25 °C and 50% RH for 48 h before testing. The initial grip separation was 20 mm, and the crosshead speed was 1 mm/s. At least six replicates were tested for each film formulation.

#### 2.4.7 Antioxidant Activity

The antioxidant activity of the films was evaluated by two methods: FRAP and TEAC. Portions of 0.5 g of the films were solubilized in 25 mL of distilled water magnetically stirred for 10 min at 60 °C.

FRAP tests were performed according to Benzie and Strain,^17^ with modifications. Solutions of acetate buffer (0.3 mol/L, pH 3.6), 2,4,6-tri­(2-pyridyl)-1,3,5-triazine (TPTZ) 10 mmol/L in HCl 40 mmol/L, and FeCl_3_ (20 mmol/L) were mixed in a proportion of 10:1:1. This mixture was a denominated FRAP reagent and was prepared immediately before starting the reaction. Of each tested sample, 20 μL of solubilized film, 180 μL of FRAP reagent, and 60 μL of distilled water were put in microplates and read in the spectrophotometer SpectraMax Mini (Molecular Devices, USA) at 595 nm after incubation at 37 °C in dark ambience for 30 min. Blank was performed in the same way, substituting the solubilized film amount for distilled water. A calibration curve (*R* ^2^ = 0.997) using Trolox as a reference was used, and the results were expressed as μmol TE/g_film_.

TEAC assays were performed according to Re, Pellegrini, Proteggente, Pannala, Yang, and Rice-Evans,^18^ with modifications. The ABTS^•+^ radical solution was prepared by mixing a 7 mM ABTS aqueous solution with a 140 mM potassium persulfate aqueous solution. The mixture was kept in a dark environment for 16 h prior to use. The ABTS^•+^ working solution was prepared by diluting the prepared mixture with ultrapure water so that the absorbance at 734 nm was 0.70 ± 0.02. For the analysis, 40 μL of solubilized films was added to 200 μL of the working radical solution in microplates. Absorbance was measured after 6 min in ambient temperature at 734 nm. Blank solutions were performed substituting the dissolved films solutions with distilled water. A calibration curve (*R* ^2^ = 0.997) using Trolox as a reference was used, and the results were expressed as μmol TE/g_film_. At least 3 replicates were performed for each formulation of film.

### 2.5 Statistical Analysis

All experiments were conducted in a completely randomized design. Data are presented as mean ± standard deviation. The data sets were subjected to analysis of variance (ANOVA) to evaluate the effect of extract concentration and polymeric matrix on the measured responses, adopting a 5% significance level (*p*-value <0.05). When applicable, significant differences identified by ANOVA were further interpreted based on the structure of the experimental factors. All statistical analyses were performed using appropriate statistical software.

## 3 Results and Discussion

### 3.1 Extraction of Phenolic Compounds

Figure 1 illustrates the sequential extraction profile obtained from passion fruit seeds, comprising two distinct stages: (i) UAE of the lipophilic fraction using absolute ethanol and (ii) subsequent UAE of phenolic compounds using 50% aqueous ethanol. This design ensures efficient removal of seed oil prior to phenolic extraction, minimizing interference from hydrophobic constituents and enhancing the solvent accessibility to phenolic-rich intracellular domains.

**Figure 1:**
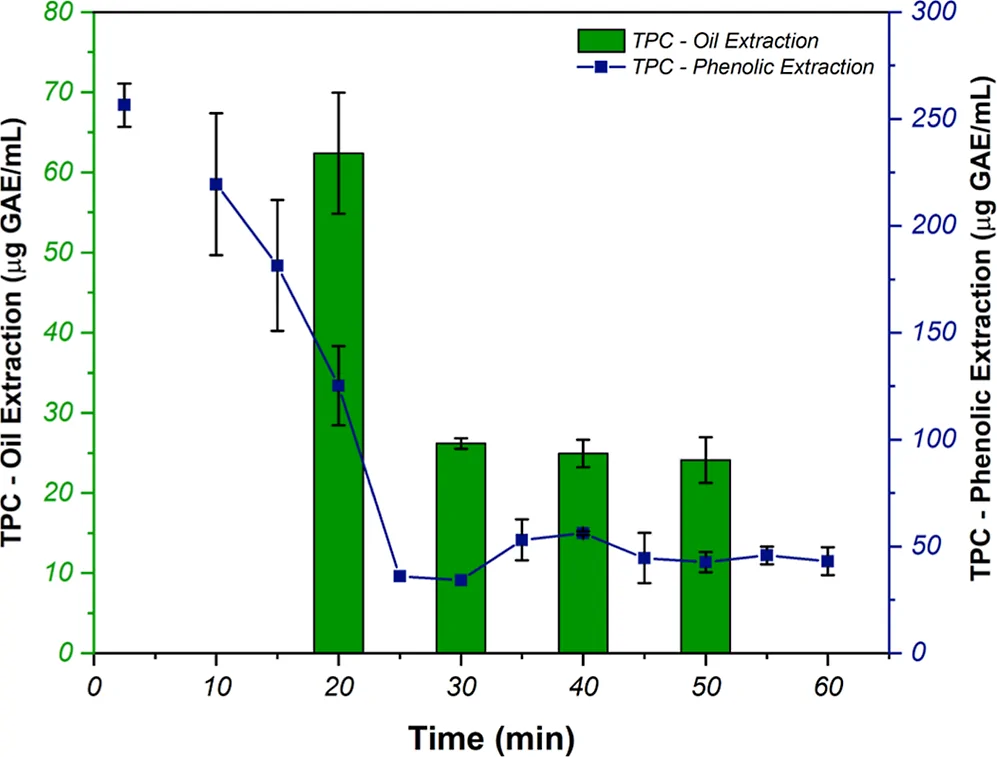
UAE of oil and phenolic compounds from passion fruit seeds.

During the first extraction stage, a rapid release of the lipid fraction is observed, reflecting the high efficiency of ethanol combined with ultrasound-induced cavitation in disrupting the oil-bearing structures. The cumulative oil extraction curve increases sharply within the initial cycles, confirming the effective defatting of the seeds under mild conditions. Once the defatted solid residue is obtained, the second extraction stage targets the phenolic compounds. A pronounced rise in TPC values occurs during the first 20 min of extraction, followed by a visible plateau. This kinetic behavior indicates that ultrasound rapidly enhances solvent penetration and cell wall disruption in the early moments, mobilizing readily accessible phenolics.^19,53^ After 20 min, the extraction rate stabilizes, suggesting depletion of the freely extractable fraction and transition to diffusion-controlled release from deeper structural regions.^20^

The pooled extract from the 2.5–20 min intervals reached 190 μg GAE/mL, confirming that passion fruit seeds, after defatting, are an excellent source of phenolic compounds. Importantly, this value is considerably higher than those typically reported for passion fruit peel obtained by conventional solvent extraction, which often requires long processing times (>180 min) to reach substantially lower TPC levels. The sequential approach used here, oil removal followed by phenolic extraction, therefore maximizes mass transfer efficiency and yields a concentrated phenolic extract, justifying its selection as the functional ingredient incorporated into the edible films.^21^

### 3.2 Visual Appearance, Thickness, and Color

All components in that study (pectin, CMC, β-glucan, glycerol, and passion fruit seed extract) are derived from food-grade sources. Pectin and CMC are generally recognized as safe (GRAS) by the FDA (21 CFR 184.1588 and 21 CFR 182.1745). β-Glucan from cereal sources has GRAS status (FDA GRN No. 739). Glycerol is GRAS (21 CFR 182.1320).^55,56^ Passion fruit seeds are consumed as a food byproduct without known toxicity. Therefore, the developed composite films are expected to be safe for contact with foods. Formal biosafety essays, including cytotoxicity and migration kinetics, are recommended prior to commercial use, which are beyond the scope of this materials characterization study. These studies should be performed in future applied research.

Although rheological characterization was not performed, visual inspection confirmed that all film-forming solutions were homogeneous, without visible phase separation or sedimentation for at least 1 h after preparation, indicating adequate dispersion stability for casting. Solvent casting remains a widely accepted method for polysaccharide film preparation in the literature.^49,50^

Figure 2 shows the visual appearance and morphology of the composite films. All formulations, both BG/CMC and BG/PEC, formed continuous, crack-free structures, indicating adequate miscibility between β-glucan and the respective structuring polymer. As the concentration of the passion fruit seed extract increased, the films exhibited a progressive transition from translucent to progressively more intense yellow-orange hues. This uniform color development across the surface suggests that the phenolic extract dispersed efficiently within both polymeric matrices, without visible pigment aggregation or phase separation. The presence of concentric color gradients in some samples reflects minor variations in local evaporation rates during solvent casting, a common phenomenon in polysaccharide films that does not compromise structural integrity.^16^

**Figure 2:**
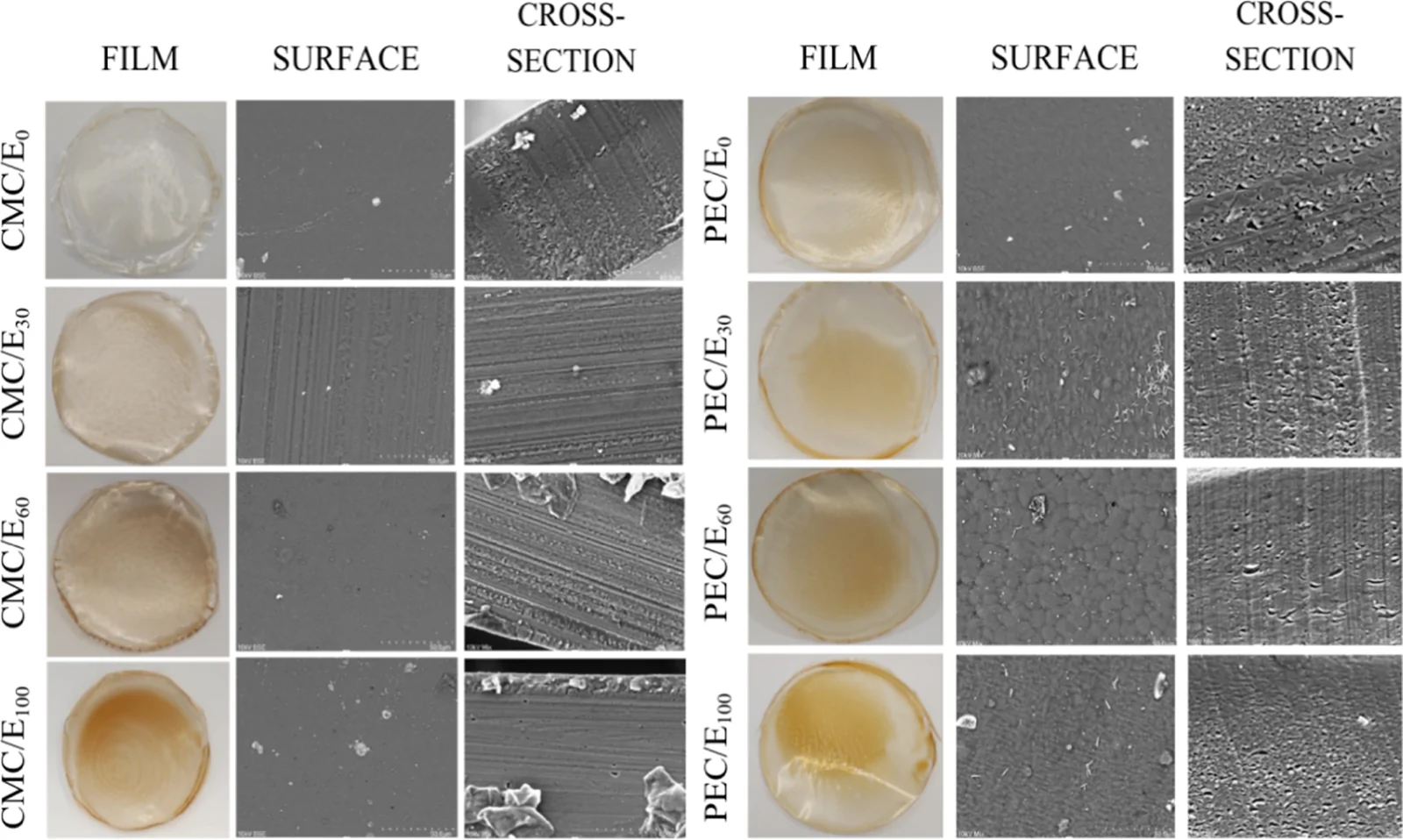
Effect of incorporating the phenolic extract from passion fruit seeds into composite β-glucan films with CMC and citrus pectin on the visual appearance and morphology.

Figure 3A presents the thickness values, which range from 0.128 to 0.158 mm, with no significant differences within each matrix (*p*-value >0.05). Although statistically similar, subtle trends were observed. In BG/CMC films, the thickness decreased slightly as the extract concentration increased, possibly due to tighter packing of polymer chains promoted by additional hydrogen bonding with phenolic compounds. In contrast, BG/PEC films showed a slight increase at the intermediate extraction level (E_60_), which may indicate localized swelling or mild plasticization arising from interactions between pectin domains and the extract. These opposite tendencies reinforce that the impact of phenolics on the film structure is dependent on the intrinsic molecular organization of each matrix.

**Figure 3:**
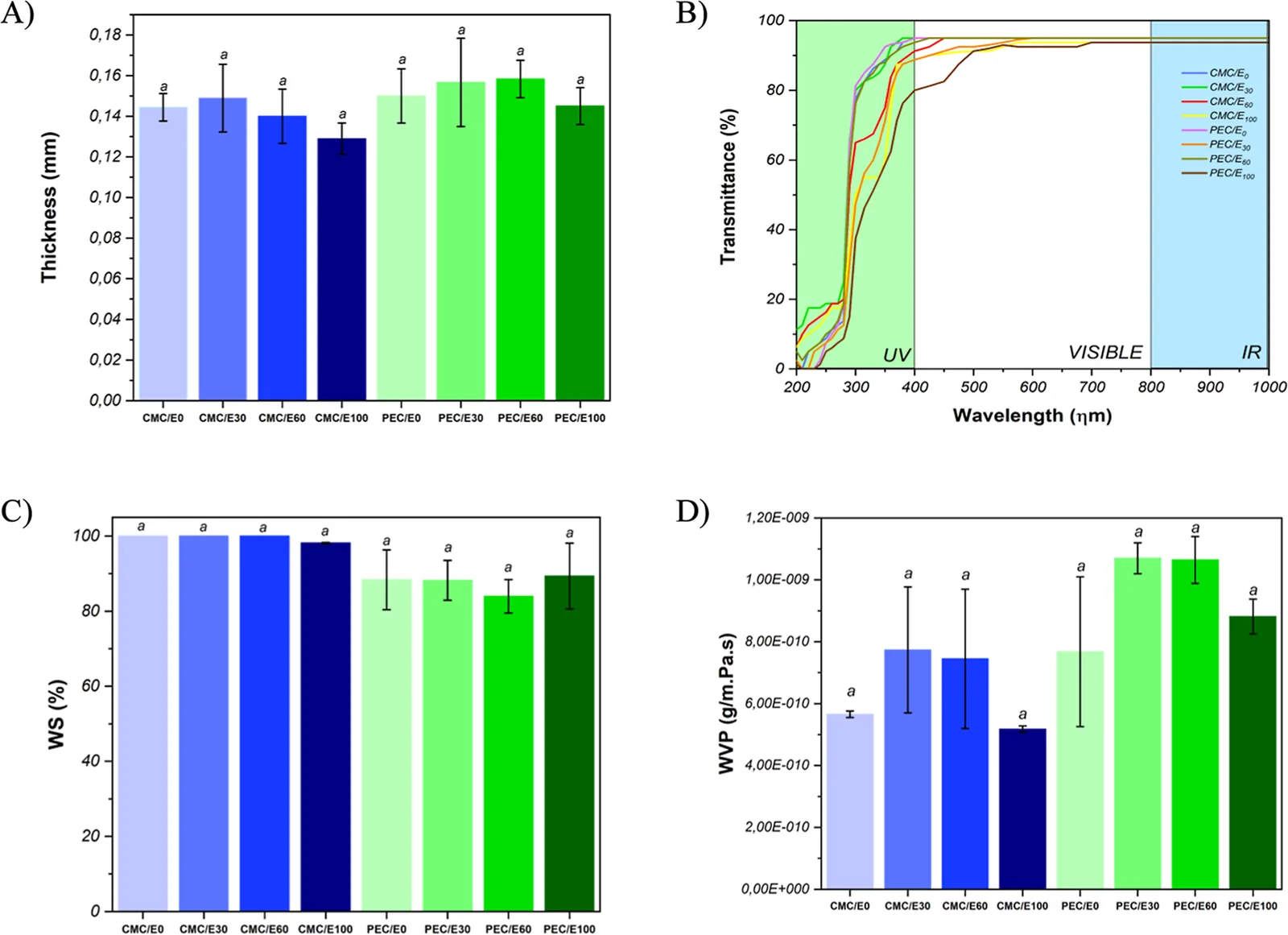
Effect of incorporating the phenolic extract from passion fruit seeds into composite β-glucan films with CMC and citrus pectin on (A) thickness; (B) light transmittance profile; (C) WS; and (D) WVP.

Colorimetric data (Table 2) support these visual assessments. Increasing extract concentration led to a consistent decline in lightness (L*) and increases in a* and especially b* values, reflecting the incorporation of the extract’s natural yellow pigments. The stronger rise in b* in BG/PEC films suggests that pectin may interact more strongly with chromophoric components of the extract, enhancing visible pigmentation. The narrow variability in color parameters within each formulation indicates a homogeneous distribution of phenolics, further confirming efficient dispersion at the macroscopic scale.

**Table 2:** Colorimetric Parameters (L*, a*, b*) of β-Glucan Composite Edible Films Formulated with CMC or Citrus Pectin

edible film	L*	a*	b*
CMC/E_0_	89.04 ± 0.25	2.03 ± 0.08	1.46 ± 0.93
CMC/E_30_	80.42 ± 1.65	4.51 ± 0.23	8.04 ± 1.81
CMC/E_60_	79.09 ± 1.52	5.19 ± 0.43	10.63 ± 1.35
CMC/E_100_	74.75 ± 5.16	7.35 ± 1.78	16.77 ± 5.31
PEC/E_0_	84.41 ± 1.26	3.34 ± 0.31	11.52 ± 2.37
PEC/E_30_	83.48 ± 1.45	3.24 ± 0.36	14.12 ± 4.12
PEC/E_60_	78.11 ± 1.87	4.88 ± 0.85	24.69 ± 3.02
PEC/E_100_	75.56 ± 0.62	5.66 ± 0.28	26.67 ± 0.59

### 3.3 Morphology and Microstructure

The microstructure of biopolymeric films is strongly influenced by the interactions among the components of the matrix, which in turn affect properties such as thickness, optical behavior, water barrier performance, TS, and EAB.^13^ The surface and cross-sectional morphology of edible films, analyzed by SEM, are presented in Figure 2. The control film CMC/E_0_ exhibited smooth, homogeneous, and compact surfaces without pores or cracks, indicating good compatibility and miscibility between β-glucan and the respective structural polymer (CMC or PEC). The cross sections of the control film showed a layered but cohesive structure. As for the pectin control film, its surface is presented as homogeneous, slightly rough and with some points indicating nonsolubilized components during the casting process. Moreover, the cross-sectional image shows higher heterogeneity, with holes indicating phase separation and the presence of layered structure.

The incorporation of the phenolic extract did not drastically alter the homogeneous morphology of the BG/CMC films, even at the highest concentration (CMC/E_100_). This suggests a strong interaction and good dispersion of the polyphenols within the CMC/BG matrix, possibly through hydrogen bonding with the numerous hydroxyl groups of the polysaccharides.^11^ In contrast, the surface of BG/PEC films became slightly rougher and more heterogeneous with the addition of the extract, showing some needle-like points at higher concentrations (PEC/E_100_, Figure 4). This could indicate crystallization of some phenolic compounds or a mild phase separation within the pectin-based matrix, which might be more sensitive to the specific interactions with the polyphenols.^22^ Finally, the cross-sectional structure of pectin films showed reduced heterogeneity with fewer holes indicating phase separation and a more subtle layered structure.

**Figure 4:**
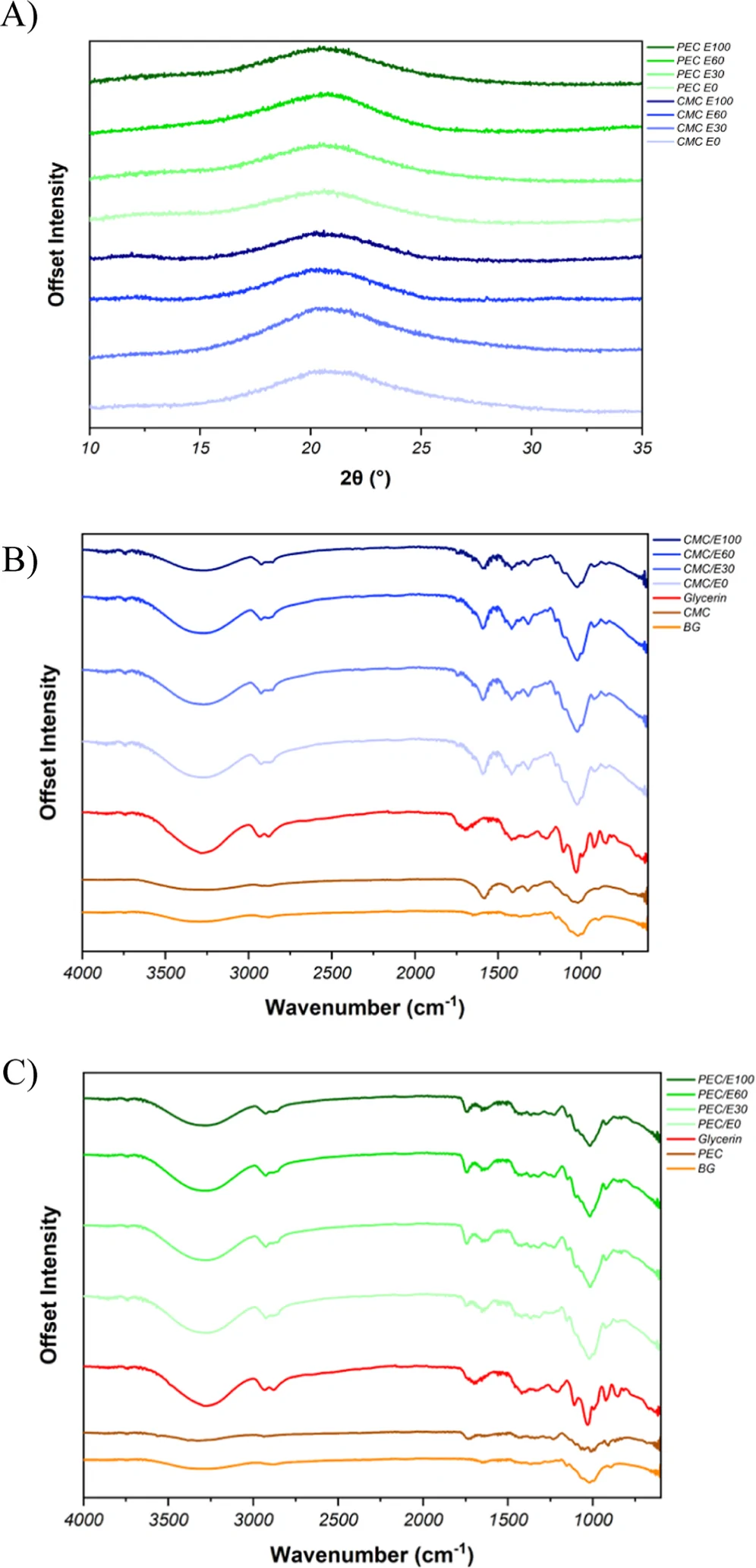
Effect of incorporating phenolic extract from passion fruit seeds into β-glucan composite films formulated with CMC or citrus pectin on (A) X-ray diffraction (XRD) patterns; (B) FTIR spectra of CMC-structured films and their individual ingredients; and (C) FTIR spectra of pectin-structured films and their individual ingredients.

### 3.4 Optical Properties and UV Barrier Capacity

The UV–vis transmission spectra of the edible films are shown in Figure 3B. All films exhibited very low transmittance in the UV region (200–320 nm), with an intense rise of the transmittance near 300 nm, indicating good UV barrier properties.^23^ This is a crucial characteristic for food packaging as UV light can catalyze lipid oxidation and cause nutrient degradation in sensitive food products. The incorporation of the passion fruit seed extract further enhanced the UV-blocking capacity. For both edible film types, increasing the extract concentration led to a decrease in the transmittance across the entire UV spectrum. This is directly attributed to the presence of light-absorbing phenolic compounds in the extract, such as flavonoids and other conjugated structures.^24^

In the visible region (400–800 nm), the control films (E_0_) showed high transmittance, confirming their transparency. The addition of the extract progressively reduced visible light transmission, imparting the characteristic yellow color observed macroscopically. Notably, the BG/PEC films generally showed a slightly lower transmittance in the visible range compared to that of BG/CMC films at equivalent extract concentrations, which correlates with their higher b* values (yellowness) shown in Table 2. Finally, the UV barrier obtained in this study corroborates that these films can be potentially used for packaging applications where light protection is desired without completely obscuring the product.

### 3.5 Water Solubility and Water Vapor Permeability

WS was prioritized over water absorption because solubility quantifies irreversible film integrity loss in humid environments, a fact corroborated by the elevated degree of solubilization for the films developed in this study. While solubility was evaluated under standard conditions, the long-term stability of the films in humid environments remained to be evaluated.

The WS of the edible films is presented in Figure 3C. The BG/CMC films were completely soluble in water (WS ∼ 100%), regardless of the extract concentration. The BG/PEC films also exhibited high solubility, ranging from 84% to 89%, which was slightly, though not significantly (*p*-value >0.05), lower than that of the CMC-based films. The high solubility is primarily due to the hydrophilic nature of the polysaccharide matrices (BG, CMC, and PEC) and the glycerol plasticizer, which facilitates water penetration and polymer dissolution.^3^ The reduction in solubility for PEC-based films, especially at intermediate extract concentrations, could be attributed to additional hydrophobic interactions or mild cross-linking induced by the polyphenols within the pectin network.^25^ This high solubility profile indicates that these films are more suitable for applications requiring rapid dissolution, such as soluble packaging or edible coatings for low-moisture products, rather than for direct contact with foods of high water activity.

We acknowledge that the high WS (∼100% for BG/CMC films and 84–89% for BG/PEC films) limits the application of these films to environments where moisture exposure is minimal or where complete disintegration is desired. Nonetheless, a high WS level is not necessarily a drawback; rather, it defines the application niche for the composite materials developed. For single-use, compostable packaging or for edible coatings on low-moisture foods (e.g., dried fruits, breads, and nuts), rapid film disintegration is advantageous as it ensures the packaging does not become a waste residue. Furthermore, several polysaccharide-based films reported in the literature exhibit similarly high solubility values (80–100%).^44–46^

The WVP results are shown in Figure 3D. The WVP values ranged from 5.18 × 10^–10^ to 1.07 × 10^–9^ g·m^–1^·s^–1^·Pa^–1^. For both film series, the incorporation of the phenolic extract at intermediate concentrations (E_30_ and E_60_) caused a significant increase (*p*-value <0.05) in WVP compared to their respective controls. This can be explained by the plasticizing effect or the disruption of the polymeric network by the polyphenols, creating more free volume and facilitating water vapor diffusion.^26^ Interestingly, at the highest extract concentration (E_100_), the WVP decreased, approaching the values of the control films. This nonlinear behavior suggests that at high concentrations, the polyphenols may act as fillers or form aggregates that create a more tortuous path for water vapor molecules, counteracting the initial plasticizing effect.^65^

From a transport phenomenon perspective, the initial WVP (E_30_, E_60_) suggests increased diffusivity due to plasticization,^41^ while the decrease at E_100_ indicates a tortuosity effect where polyphenol aggregates hinder water vapor diffusions. The nonlinear WVP behavior as a function of extract concentration is consistent with the filler effect described for other polysaccharide-phenolic systems.^42^

To provide a quantitative interpretation of the WVP behavior, the solution-diffusion model was applied,^40^ where WVP = *S* × *D* (*S* = water solubility of the water vapor in the film; *D* = diffusivity). Moreover, assuming the hypothesis that *S* is constant for every formulation—a valid assumption given the hydrophilic tendency of both the polysaccharides and the phenolic extract—changes in WVP are attributed to changes in *D*. The relative diffusivity (*D*_E0_/*D*_Ei_) was estimated for each formulation (Table 3).

**Table 3:** Relative Diffusivity (*D*_rel_) of β-Glucan (BG) Composite Films Containing Carboxymethylcellulose (CMC) or Pectin (PEC) and Different Concentrations of Phenolic Extract

formulation	WVP (10^–10^)	*D*_rel_
CMC/E_0_	5.655	1.000
CMC/E_30_	7.735	1.368
CMC/E_60_	7.450	1.317
CMC/E_100_	5.180	0.916
PEC/E_0_	7.680	1.000
PEC/E_30_	10.700	1.390
PEC/E_60_	10.645	1.386
PEC/E_100_	8.815	1.148

For BG/CMC films, the relative diffusivity (*D*_rel_) increased from 1.00 (E_0_) to 1.37 (E_30_) and 1.32 (E_60_), indicating an augment in the diffusivity by 36.8% and 31.7%, respectively, due to polymer chain disruption (plasticization). At E_100_, *D*_rel_ decreased to 0.92 (8%), approaching the control value. Similarly, the GB/PEC films exhibited the same pattern for *D*_rel_: increase of relative diffusivity at E_30_ and E_60_ (∼39% on both) and a decrease at E_100_ (15%). The decrease of *D*_rel_ between E_60_ and E_100_ indicates that the diffusion path length increased, likely due to the presence of phenol aggregates that act as obstacles for the mass transport phenomena.^42,43^

Overall, the BG/CMC films exhibited slightly lower WVP values than the BG/PEC films, which is consistent with the more compact and homogeneous morphology observed by SEM. Despite the variations, the WVP values obtained are within the range reported for other polysaccharide-based edible films.^3,26^

### 3.6 X-ray Diffraction

The X-ray diffractograms of the edible films are presented in Figure 4A. All films showed broad halos with no sharp crystalline peaks, indicating a predominantly amorphous structure. This is typical for polysaccharide-based films plasticized with glycerol, where the molecular chains are arranged in a disordered manner.^27^ For the BG/CMC films, the incorporation of the phenolic extract at low concentration (E_30_) caused a slight increase in the intensity of the amorphous halo centered around 20°. However, higher extract concentrations (E_60_, E_100_) led to a reduction in intensity. For the BG/PEC films, a gradual increase in amorphous halo intensity was observed with increasing extract concentration. These subtle changes in the XRD patterns suggest that the phenolic compounds interact with the polymeric chains, affecting their short-range order and packing density. The absence of new crystalline peaks confirms that the polyphenols were molecularly dispersed within the matrix and did not recrystallize on a macroscopic scale, maintaining the generally amorphous structure, which aligns with the generally homogeneous morphology seen in SEM.^28^

### 3.7 Fourier-Transform Infrared Spectroscopy

The FTIR spectra confirmed the successful incorporation of all components and revealed the main interactions within the edible film matrices. The spectra of BG/CMC films (Figure 4B) showed characteristic broad bands, a strong band at ∼3300 cm^–1^ (O–H stretching), bands at ∼2900 cm^–1^ (C–H stretching), a band at ∼1590 cm^–1^ (asymmetric –COO^–^ stretching of CMC), and a band at ∼1026 cm^–1^ (C–O–C stretching of glycosidic bonds).^4,29^ The incorporation of the phenolic extract did not cause peak shifts but led to a gradual reduction in the intensity of the O–H and C–O–C bands. This attenuation is likely due to the overlapping of the polysaccharide signals with those of the polyphenols and to increased hydrogen bonding interactions, which can broaden and reduce band intensity.^3^

The spectra of BG/PEC films (Figure 4C) displayed similar features, with additional characteristic bands of pectin, a shoulder at ∼1739 cm^–1^ (C=O stretching of esterified carboxyl groups), and a band at ∼1650 cm^–1^ (asymmetric stretching of free carboxylate groups). The intensity of the ester band (1739 cm^–1^) increased relative to the carboxylate band with higher extract concentrations, suggesting potential interactions (hydrogen bonding) between the phenolic hydroxyl groups and the pectin’s carbonyl/carboxylate groups, which might slightly affect the local environment of these functional groups. No new peaks appeared, indicating the absence of covalent chemical bonds and confirming that the interactions are primarily physical.^30^ Also, the ingredients used showed the same common bands as the films (∼3300 cm^–1^, O–H stretching; ∼2900 cm^–1^, C–H stretching; ∼1590 cm^–1^, asymmetric –COO^–^ stretching of CMC; and ∼1026 cm^–1^, C–O–C stretching of glycosidic bonds), indicating good synergy between the polymers in the matrix.

### 3.8 Thermogravimetric Analysis

While differential scanning calorimetry (DSC) was not available, derivative thermogravimetry (DTG) provided quantitative thermal degradation data, including peak degradation temperatures and mass loss rates, which are complementary to those of DSC for evaluating polymer miscibility and thermal stability. DTG allows distinguishing peak degradation temperatures, mass loss rates, and the temperatures at which degradation begins and finishes.

The thermal stability of the edible films was evaluated by thermogravimetric analysis (TGA) as presented in Figures 5 and 6. All films exhibited three main stages of weight loss: (i) up to 150 °C (loss of free and bound water), (ii) 200–350 °C (major degradation of polysaccharide backbones and glycerol), and (iii) above 350 °C (carbonization of residues). The BG/CMC films (Figure 5) showed higher thermal stability than the BG/PEC films (Figure 6), with the main decomposition step occurring at higher temperatures. This is consistent with the intrinsic thermal behavior of the raw materials, where CMC is generally more thermally stable than citrus pectin.^31^

**Figure 5:**
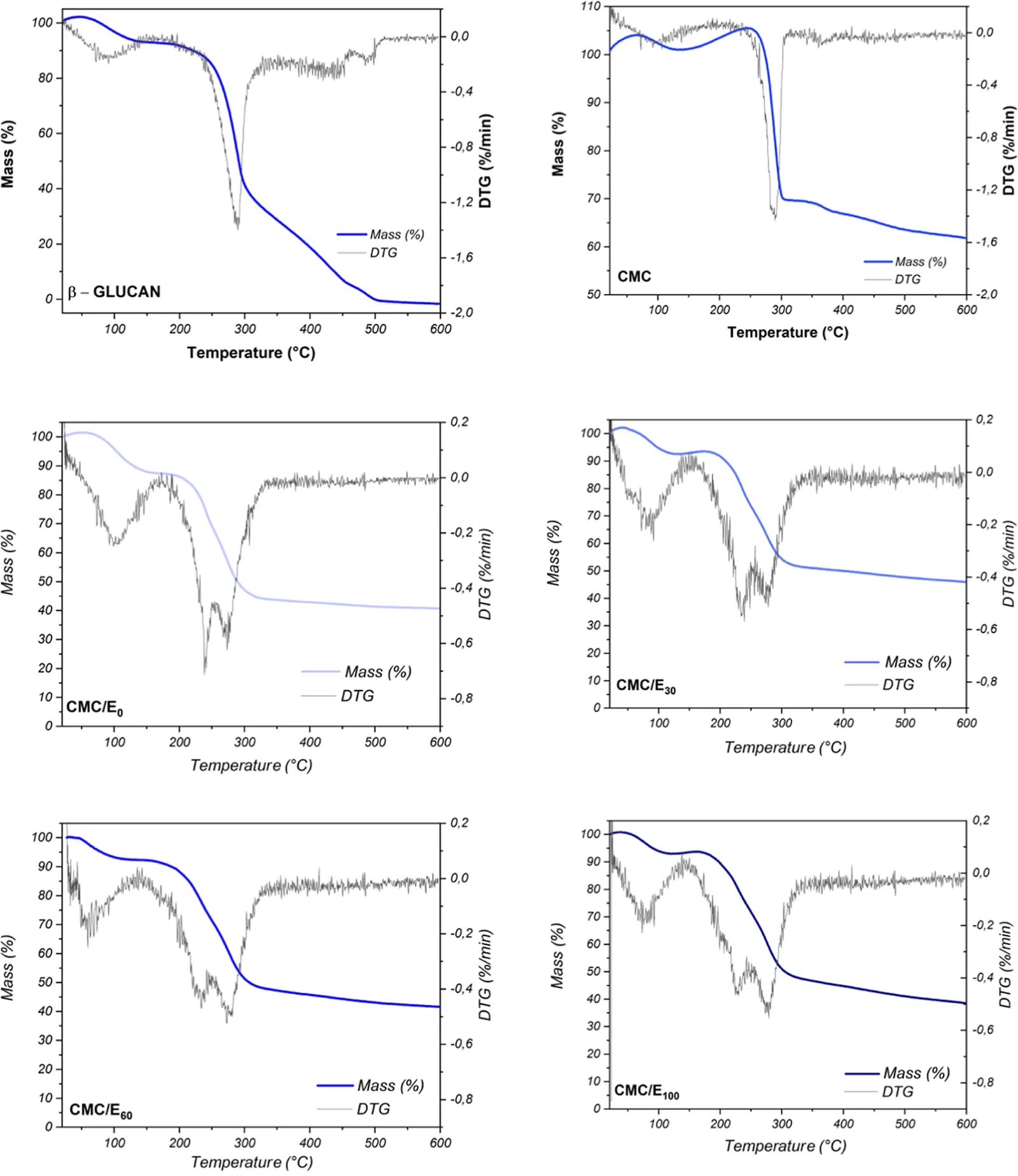
Effect of incorporating phenolic extract from passion fruit seeds into β-glucan composite films formulated with CMC on their thermal stability, as assessed by TGA, including comparison with the individual CMC and β-glucan ingredients.

**Figure 6:**
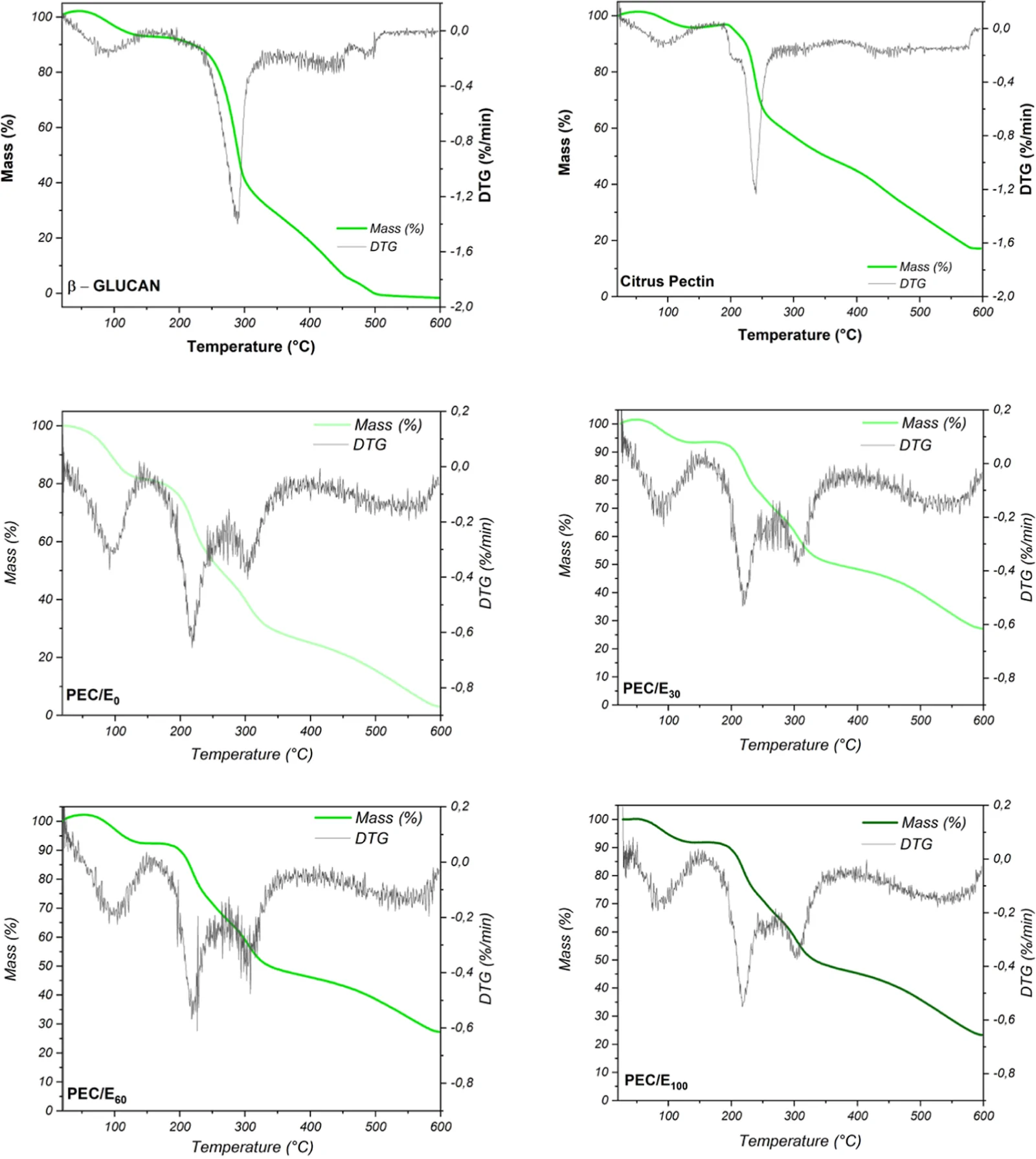
Effect of incorporating phenolic extract from passion fruit seeds into β-glucan composite films formulated with citrus pectin on their thermal stability, as assessed by TGA, including comparison with the individual citrus pectin and β-glucan ingredients.

The incorporation of the phenolic extract positively influenced the thermal stability of both edible film types. Compared to the controls (E_0_), films containing the phenolic extract showed a reduction in the maximum mass loss rate and a slight shift of the main degradation step to higher temperatures, especially noticeable in the BG/PEC films. The most significant protective effect was observed at intermediate extract concentrations (E_60_). Quantitative analysis of the DTG thermograms revealed that the main degradation peak temperature for the BG/CMC did not shift considerably from the range 240–280 °C revealed by the double peaks in the control film (E_0_). However, there was a significant decrease in the mass loss rate (d*W*/d*T*_max_) of 14.6% and 2.3% for E_30_ and E_60_ and an increase of 2.3% for E_100_, indicating that in intermediate concentrations, the phenolic extract acts as a thermal stabilizer in CMC composite films, likely due to the hydrophilic groups present in the polyphenol extract.^48,59^

As for the BG/PEC films, there was a significant decrease in the mass loss rate for all films formulated with phenolic extract from passion fruit seeds: 30% for the E_30_ and E_60_ films and 25% for the E_100_ film. Once again, the behavior of the mass loss rate in the function of the extract concentrations is similar to that of the BG/CMC films, though considerably more influential. No major shifts from the original maximum degradation temperature peaks were observed.^47,48^

This improvement in thermal stability can be attributed to the antioxidant activity of the polyphenols, which may scavenge free radicals generated during heating, delaying the onset of polymer chain scission. Furthermore, the interactions (hydrogen bonding) between the polyphenols and the polymer matrices could restrict chain mobility, providing additional thermal stability.^32^

The thermal degradation parameters, including onset temperature (*T*_0_), offset temperature (*T*_f_), and char yield at 600 °C, are summarized in Table 4. The BG/CMC films exhibited higher *T*_0_ and *T*_f_ values compared to BG/PEC films, confirming the intrinsically higher thermal stability of CMC-based matrices, consistent with the thermal behavior of the raw materials.^31^ For the BG/CMC series, the onset temperature (*T*_0_) ranged from 103.29 °C (CMC/E_0_) to 89.94 °C (CMC/E_100_), exhibiting a slight decrease upon extract incorporation. The offset temperature (*T*_f_) remained relatively stable at approximately 337 °C for all formulations, indicating that the extract did not significantly alter the completion temperature of the main degradation event. The char yield at 600 °C ranged from 38.62% (CMC/E_100_) to 45.99% (CMC/E_30_), demonstrating that CMC-based films form a substantial carbonaceous residue, characteristic of cellulose derivatives.^47,48^

**Table 4:** Thermal Degradation Parameters of β-Glucan (BG) Composite Films Containing Carboxymethylcellulose (CMC) or Pectin (PEC) and Different Concentrations of Phenolic Extract: Onset Temperature (*T*_0_, °C), Offset Temperature (*T*_f_, °C), and Char Yield at 600 °C

formulation	*T*_0_ (°C)	*T*_f_ (°C)	char yield (%) at 600 °C
CMC/E_0_	103.29	337.37	40.74
CMC/E_30_	97.32	336.97	45.99
CMC/E_60_	80.03	337.64	41.63
CMC/E_100_	89.94	337.10	38.62
PEC/E_0_	74.91	360.89	3.02
PEC/E_30_	109.65	356.28	27.15
PEC/E_60_	112.82	350.33	27.29
PEC/E_100_	97.86	352.71	23.28

In contrast, the BG/PEC films showed a more pronounced response to the phenolic extract. The onset temperature (*T*_0_) increased from 74.91 °C (PEC/E_0_) to 112.82 °C (PEC/E_60_), indicating that the extract delayed the onset of thermal degradation. The most striking effect was observed in the char yield: the control pectin film (PEC/E_0_) presented a negligible char yield of only 3.02%, while the incorporation of the phenolic extract considerably increased this value to approximately 27% for PEC/E_30_ and PEC/E_60_, with a slight reduction to 23.28% at the highest concentration (PEC/E_100_). This substantial increase in residual mass suggests that the phenolic compounds promote char formation, likely through cross-linking reactions and the formation of stable carbonaceous structures during heating.^47,48^

The different behaviors of the two matrices highlight the role of polymer–polyphenol interactions in determining thermal stability. The pronounced effect of the extract on pectin-based films may be attributed to the higher affinity of phenolic compounds for pectin’s carboxyl and hydroxyl groups, facilitating hydrogen bonding and promoting a more thermally stable network.^32,47^ The reduction in char yield at the highest extract concentration (E_100_) for both film series may indicate that excess polyphenols disrupt the polymer network, partially counteracting the protective effect observed at intermediate concentrations.^48^

### 3.9 Mechanical Properties

The mechanical properties of edible films are critical for their handling and application as packaging. The TS and EAB of the composite films are presented in Figure 7A,B. The control films exhibited distinct mechanical behaviors based on their polymeric matrix. The BG/PEC control (PEC/E_0_) showed a higher TS (3.33 ± 0.24 MPa) but lower EAB (11.96 ± 2.37%) compared to the BG/CMC control (CMC/E_0_, TS: 3.12 ± 0.19 MPa and EAB: 20.71 ± 3.11%). This indicates that the BG/PEC matrix forms a stronger but more brittle network, while the BG/CMC matrix is more flexible and ductile. The higher flexibility of CMC-based films is often attributed to the linear and flexible chains of CMC and its interaction with BG, allowing greater chain mobility under stress.^33^

**Figure 7:**
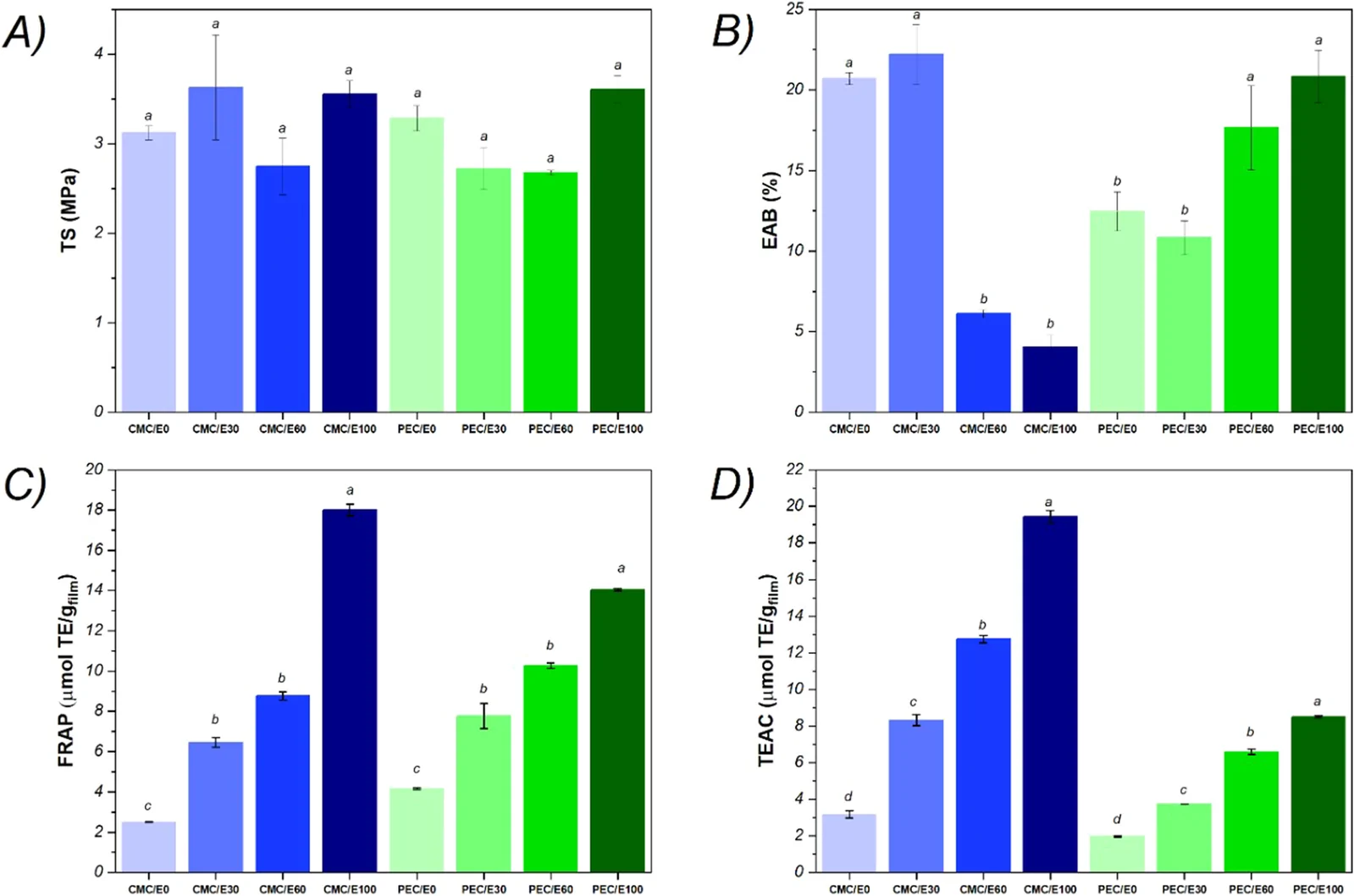
Effect of incorporating phenolic extract from passion fruit seeds into β-glucan composite films formulated with CMC or citrus pectin on (A) TS; (B) EAB; (C) antioxidant activity determined by the FRAP assay; and (D) antioxidant activity determined by the TEAC assay.

The incorporation of the passion fruit seed extract had a significant and matrix-dependent effect on the mechanical properties. For the BG/CMC films, a low-concentration extract (E_30_) slightly increased both TS and EAB, suggesting that a small amount of polyphenols may act as a compatibilizer or mild cross-linker within the matrix. However, higher extract concentrations (E_60_ and E_100_) led to a drastic reduction in TS and EAB, making the films more brittle.^34^ This is likely due to an excess of polyphenols disrupting the continuous polysaccharide network, creating stress concentration points, and reducing the film’s ability to deform plastically.^35^

In contrast, for the BG/PEC films, the effect was the opposite and more favorable. While the low-concentration extract (E_30_) caused a slight reduction in mechanical properties, the intermediate- and high-concentration extracts (E_60_ and E_100_) progressively improved both TS and, most notably, EAB. The film PEC/E_100_ achieved the best-balanced mechanical performance in this series, with a TS of 3.64 MPa and an EAB of 20.84%. This significant enhancement in flexibility suggests that at optimal concentrations, the polyphenols in the pectin-based matrix may act as effective plasticizers or interact in a way that increases the cohesion and extensibility of the film network without sacrificing strength.^36^ These divergent results highlight the importance of the polymer matrix in determining the final effect of an active compound. The optimal formulation for mechanical performance was CMC/E_30_ for the CMC series and PEC/E_100_ for the pectin series.

### 3.10 Antioxidant Activity

The primary functional objective of incorporating the passion fruit seed extract was to provide antioxidant activity for the edible films. In active packaging systems, such antioxidant functionality, often combined with antimicrobial effects when plant-based extracts are used, can contribute to delaying oxidative degradation, extending food shelf life, and ultimately reducing organic waste.^37^ The results of the FRAP and TEAC assays are presented in Figure 7C,D.

The control films (E_0_) exhibited a low but measurable base-antioxidant activity. This can be attributed to the intrinsic antioxidant capacity of the biopolymers themselves, particularly the commercial citrus pectin, which may contain residual bioactive compounds from its source.^38^ The BG/PEC control showed slightly higher activity than the BG/CMC control.

A strong, concentration-dependent increase in antioxidant activity was observed for all films incorporating the phenolic extract. The results from both the FRAP (reducing power) and TEAC (radical scavenging) assays were highly correlated, confirming the reliability of the measurements.^66^ For the BG/CMC films, the antioxidant activity increased dramatically, reaching 19.42 ± 1.15 μmol TE/g (TEAC) for the CMC/E_100_ formulation. For the BG/PEC films, the increase was also significant but reached a slightly lower plateau of 14.04 ± 0.87 μmol TE/g (TEAC) for PEC/E_100_.

The more pronounced increase in the BG/CMC films suggests a potentially higher accessibility or more efficient release of the phenolic compounds from the CMC matrix during the assay. CMC’s high solubility capacity likely facilitates the rapid diffusion of antioxidants into the assay medium. The values obtained are comparable to or higher than those reported for other active films incorporated with fruit byproduct extracts, underscoring the high antioxidant potential of passion fruit seed phenolics.^39^ These results demonstrate the successful development of active packaging films. The dose-dependent antioxidant power allows for tailoring the film’s activity based on the required shelf life extension for specific oxygen-sensitive food products.

## 4 Conclusions

This study demonstrated a comprehensive and sustainable strategy for converting yellow passion fruit seed waste into high-value functional ingredients and active edible films. By integrating a sequential UAE with β-glucan-based composite matrices, we established how the polymeric architecture critically dictates the functional response of active films, revealing clear matrix-dependent behaviors. Although both β-glucan/CMC and β-glucan/pectin films successfully incorporated the phenolic extract and exhibited amorphous, thermally stable structures, their physicochemical and mechanical performances diverged significantly due to differences in polymer–polyphenol interactions.

β-Glucan/CMC films showed high solubility (∼100% for β-glucan/CMC films and 84–89% for β-glucan/pectin films), enhanced UV-blocking capacity, and the highest antioxidant potential, reaching 19.42 μmol TE/g by TEAC assay at the maximum extract loading, confirming their suitability for applications where rapid film dissolution and intense antioxidant release are desired. In contrast, β-glucan/pectin films demonstrated a superior structural organization and mechanical reinforcement upon extract incorporation, achieving an optimal balance between TS (3.64 MPa) and EAB (20.84%) at high extract concentration (β-glucan/pectin films), while the optimal balance was achieved at intermediate/low extract contents for β-glucan/CMC films (3.65 MPa, 22.5%).

These opposite trends highlight that the same bioactive extract can plastify, disrupt, or reinforce biopolymer networks, depending on the governing matrix. Together, the results establish a versatile platform in which the functional profile of the edible films can be tailored by selecting the appropriate polysaccharide matrix, enabling targeted applications ranging from high-integrity mechanical packaging to fast-dissolving antioxidant carriers. This work advances circular bioeconomy principles by demonstrating that passion fruit processing waste can be upcycled into tunable, high-performance materials for active food packaging. The present study focused exclusively on the physical, mechanical, thermal, structural, and barrier characterization of the developed composite films. While passion fruit seed phenolic compounds are reported in the literature to possess antimicrobial activity against food-related pathogens (e.g., *Escherichia coli*, *Staphylococcus aureus*, *Bacillus subtilis*, and *Clostridium albicans*), such determinations require biosafety facilities, which were not available during the execution of this study and were beyond the scope of this materials-focused paper.

Although dip-coating trials on fresh-cut fruits/vegetables would be valuable for demonstrating real applicability, controlled storage facilities and daily visual documentation over extended periods were not available. Likewise, aging studies under varying environmental conditions were not performed according to the mentioned limitations. Future investigations should apply these optimized formulations to real food systems, assess release kinetics under practical conditions, explore synergistic functionalities such as antimicrobial activity or controlled degradation behavior, and evaluate cross-linking strategies in order to enhance the film properties.
